# Biological Evaluation and Potential Applications of Secondary Metabolites from Fungi Belonging to the Cordycipitaceae Family with a Focus on *Parengyodontium* spp.

**DOI:** 10.3390/jof11110764

**Published:** 2025-10-24

**Authors:** Dylan Marin, Philippe Petit, Ludovic Pruneau

**Affiliations:** ECOTERCA (ÉCOlogie TERrestre CAribéenne), Faculté des Sciences Exactes et Naturelles, Université des Antilles, Campus de Fouillole, BP 592, 97159 Pointe-à-Pitre, France; dylan.marin@etu.univ-antilles.fr (D.M.); philippe.petit@univ-antilles.fr (P.P.)

**Keywords:** *Parengyodontium*, *Cordycipitaceae*, secondary metabolites

## Abstract

Fungi of the genus *Parengyodontium* (Ascomycota, Hypocreales, Cordycipitaceae) are emerging as promising sources of secondary metabolites with significant biotechnological potential. While traditionally understudied, species such as *Parengyodontium album*, *Parengyodontium torokii* and *Parengyodontium americanum* have been isolated from diverse and sometimes extreme environments—including deep-sea sediments, mangroves, and NASA clean rooms—suggesting remarkable ecological adaptability. This review presents a comprehensive synthesis of current knowledge on the chemical diversity, biological activities, and potential industrial applications of secondary metabolites produced by fungi belonging to the genus. A wide variety of compounds have been identified, including polyketides (e.g., engyodontiumones, alternaphenol B2), terpenoids (e.g., cytochalasin K), alkaloids, and torrubielline derivatives. These metabolites exhibit cytotoxic, antibacterial, and antifouling properties, with promising anticancer and antimicrobial activities. In addition, recent evidence points to the genus’s role in bioremediation, particularly through the degradation of polyethylene by *P. album*. Despite the advances highlighted here, challenges remain in scaling production, elucidating biosynthetic pathways, and confirming in vivo efficacy. This review underscores the value of integrating chemical, genomic, and metabolomic approaches to fully unlock the biotechnological potential of *Parengyodontium* species. Additionally, we broaden the perspective by comparing trends in secondary metabolites among Cordycipitaceae, highlighting lifestyle-related chemical compounds that serve as a reference for the *Parengyodontium* profile.

## 1. Introduction

Fungi represent an invaluable reservoir of secondary metabolites, which are both structurally diverse and biologically active, many of which have become key molecules in medicine, agriculture, and biotechnology [1,2]. Unlike primary metabolites, secondary metabolites are not essential for the immediate survival of the microorganism, but offer an adaptive advantage in competitive or stressful environments, acting in particular as antimicrobial agents, signaling molecules, or defense compounds [3,4,5]. To date, most research has focused on prolific fungal genera such as *Penicillium*, *Fusarium*, and *Aspergillus*, leading to the discovery of numerous bioactive molecules such as penicillin, lovastatin, and fusaric acid [6]. However, there is now growing interest in fungal taxa that have been little studied to date, particularly those from extreme or atypical habitats, whose biosynthetic pathways could lead to the production of novel metabolites [7,8,9,10,11].

The Cordycipitaceae family (*Hypocreales*, *Sordariomycetes*) mainly comprise entomopathogenic fungi that infect insects and spiders, but also include mycoparasitic, endophytic, and lichenicolous lineages. Their modern circumscription is based on multilocus phylogenies (nrSSU, nrLSU, TEF1-α, rpb1/2) that have harmonized teleomorphic and anamorphic nomenclature and stabilized generic usage [12,13]. The core of the family currently includes *Cordyceps*, *Beauveria*, *Akanthomyces*, *Samsoniella*, *Gibellula*, *Hevansia*, *Blackwellomyces*, *Simplicillium* and *Leptobacillium*, as well as hyphomycetes such as *Parengyodontium*; the taxonomy remains dynamic, with the resurrection of *Lecanicillium* and the creation of related genera (*Corniculantispora*, *Corpulentispora*, *Zarea*, *Zouia*) [14]. Recent syntheses estimate the current number of recognized genera at around 38, reflecting sustained diversification supported by molecular systematics [12,13,14,15,16,17,18].

**Figure 1:**
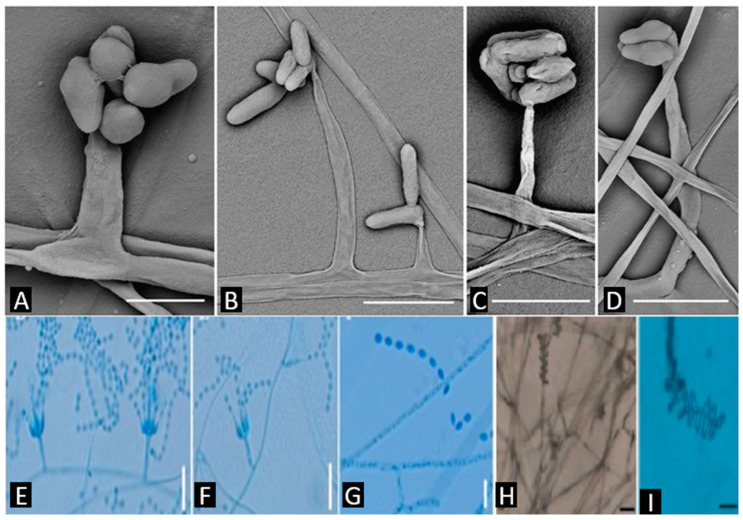
*Cordyceps bullispora*, (**A**–**D**) phialides [19]. *Samsoniella coccinellidicola*, (**E**,**F**) conidiogenous cells (conidiophores, phialides), (**G**) conidia on Potato Dextrose Agar (PDA) [20]. *Blackwellomyces kaihuaensis*, (**H**,**I**) conidia [21]. Scale bars: (**A**) 5 µm; (**B**) 15 µm; (**C**–**I**) 10 µm. Source of images: panels (**A**–**D**) reproduced from [19]; (**E**–**G**) from [20]; (**H**,**I**) from [21] (reproduced/adapted from previously published works as cited).

The Cordycipitaceae comprise several morphologically coherent sets of genera. First are the cordycipitoid teleomorphs with colored stromata *Cordyceps* [19] (Figure 1), *Samsoniella* [20] (Figure 1), and *Blackwellomyces* [21] (Figure 1). These fungi form stipitate, orange-to-red stromata with immersed to sub-immersed perithecia. Ascospores are filiform and typically disarticulate into part-spores in *Cordyceps* and *Samsoniella*, whereas in *Blackwellomyces* they remain non-disarticulating, a critical diagnostic separating it from its look-alikes [12,13,22]. Hosts are mainly insects and other arthropods.

**Figure 2:**
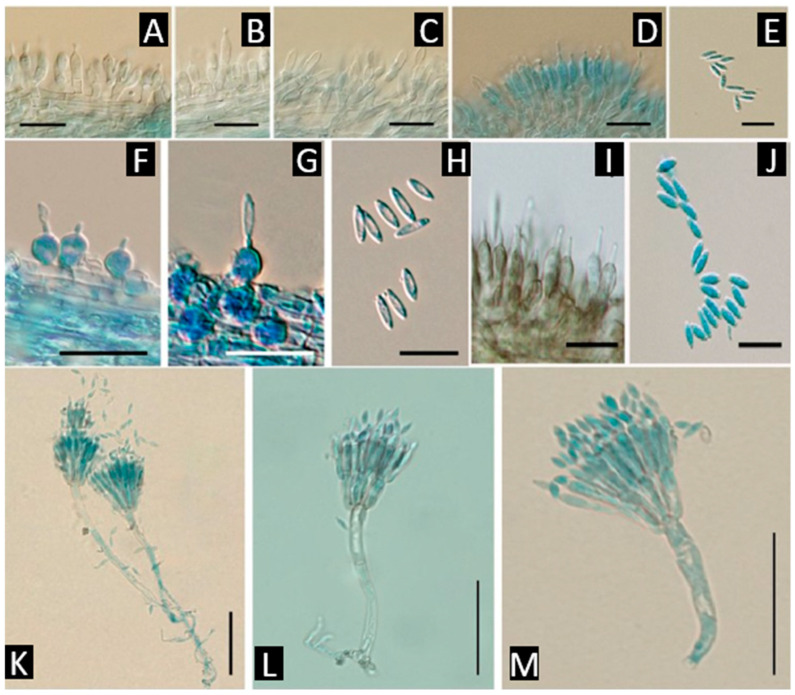
*Akanthomyces noctuidarum*, (**A**–**C**) phialides through the length of synnema, (**D**) phialides at the tip of synnema, (**E**) conidia [23]. *Hevansia novoguineensis*, (**F**–**H**) phialides with conidia on synnema [18]. *Jenniferia griseocinerea*, (**I**) phialides, (**J**) conidia [18]. *Gibellula penicillioides*, (**K**–**M**) penicillate conidiophores [24]. Scale bars: (**A**–**J**) 10 µm; (**K**–**M**) 50 µm. Sources of images: panels (**A**–**E**) reproduced from [23]; (**F**–**J**) from [18]; (**K**–**M**) from [24] (reproduced/adapted from previously published works as cited).

A second cluster encompasses isarioid hyphomycetes with synnemata, notably *Akanthomyces* [23] (Figure 2) and the spider-associated *Hevansia* [18] (Figure 2) (with related segregates such as *Jenniferia* [18] (Figure 2) and *Parahevansia*). These taxa produce cylindrical synnemata sheathed by hymeniform layers of phialides that yield hyaline conidia in chains; many species parasitize insects and spiders [12,22,23]. A close but readily distinguished lineage is isarioid forms with aspergilliform heads, i.e., *Gibellula* [24] (Figure 2), whose whitish synnemata or cushions bear metulae plus phialides forming aspergilliform/penicillioid heads, an architecture that separates it from *Akanthomyces*/*Hevansia*. *Gibellula* is characteristically spider-pathogenic [24,25].

**Figure 3:**
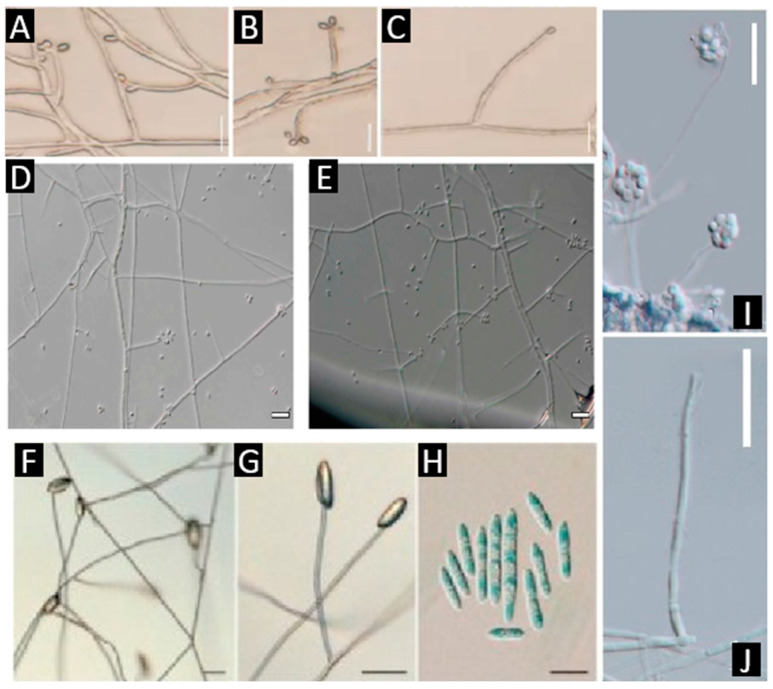
*Beauveria polyrhachicola*, (**A**–**C**) Conidiogenous cells and conidia [28]. *Parengyodontium torokii*, (**D**) Conidia produced at each bent point of the zigzag rachides of the fertile conidiogenous cells, (**E**) Whorl of two conidiogenous cells with conidi attached at the zigzag rachides [29]. *Ascopolyporus albus*, (**F**,**G**) phialide and conidia on PDA, (**H**) conidia on PDA [32]. *Simplicillium formicae*, (**I**,**J**) phialides bearing conidia [31]. Scale bars: (**A**–**C**,**H**,**J**) 10 µm; (**D**–**G**) 20 µm; (**I**) 30 µm. Source of images: panels (**A**–**C**) reproduced from [28]; (**D**,**E**) from [29]; (**F**–**H**) from [32]; (**I**,**J**) from [31] (reproduced/adapted from previously published works as cited).

A third morphological block includes hyaline hyphomycetes without synnemata, which split into three practical profiles. The «zig-zag» type, *Beauveria*, shows sympodial zig-zag conidiogenesis along a denticulate rachis and produces dry, hydrophobic, powdery conidia [22,26,27,28]. The «rachis with denticles (no zig-zag)» type (*Parengyodontium*, Figure 3) forms conidiophores as straight rachides studded with denticles; colonies are typically cottony white, and species are often environmental or opportunistic [29]. Finally, the minimalist hyaline profile (*Simplicillium*, Figure 3) features very slender, often solitary phialides producing small mucoid heads and lacking synnemata; many species are entomopathogens, endophytes, or mycoparasites [30,31].

A fourth, contrasting set comprises taxa with perithecia naked on a subiculum, the “torrubielloid” look exemplified by *Ascopolyporus* [32] (Figure 3). Here, perithecia are superficial and aggregated on a felted mycelial mat that coats host plants (often grasses or bamboo), and hosts are typically scale insects (Hemiptera). The absence of a fleshy stroma and the «felted crust + naked perithecia» habit place these fungi far from the cordycipitoid stromatal morphology and make them easy to recognize in the field [12,22].

Together, these groupings show how a handful of characters stromatal architecture, ascospore behavior (disarticulating vs. non-disarticulating), conidiogenesis (zig-zag vs. denticles vs. solitary phialides), presence or absence of synnemata, and host specialization (insects vs. spiders vs. scale insects) allow rapid, morphology-based placement at genus level across Cordycipitaceae, with molecular phylogenies providing the backbone for difficult cases [12,13,22].

The genus *Parengyodontium*, belonging to the family Cordycipitaceae (*Hypocreales*, *Ascomycota*), comprises filamentous ascomycete fungi. Formerly included in the genera *Engyodontium* or *Tritirachium*, recent multi-locus genetic analyses have made it possible to redefine the species known today as *Parengyodontium* spp. [33]. Currently, there are three described species: *Parengyodontium album*, *Parengyodontium torokii*, and *Parengyodontium americanum*. These fungi are highly adaptable due to the diversity of the environments from which they have been isolated [34]. *P. torokii* was found in the assembly building of NASA’s Mars 2020 mission [29]. *P. album* has been isolated from plants [35,36,37], soils [38,39] and marine environments [40,41,42,43,44]. *P. americanum* has been isolated from cultures of fungi of the genus *Coccidioides* [45]. Additionally, beyond natural and industrial habitats, *P. album* is a frequent colonizer of cultural heritage environments, where it has been repeatedly reported on stone, plaster/brick, glass, paper and painted surfaces (including wall paintings) across historic and religious buildings, monuments, museums and libraries [46,47,48]. Although cultural heritage itself is not the focus of this review, the breadth of substrates and niches remains relevant, as it underscores the species’ ecological versatility and surface-associated lifestyles that likely shape enzyme repertoires and condition-dependent secondary metabolism [34,48,49].

In addition to their remarkable distribution, species of the genus *Parengyodontium* attract attention for their dual potential: the biosynthesis of secondary metabolites of interest and their application in bioremediation. They are a major source of bioactive molecules used in the pharmaceutical, agrochemical, and environmental fields [42,44,50,51,52,53,54,55,56]. Although these compounds are not essential for fungal growth, they play a fundamental role in its environmental interactions and therefore have considerable potential for application. In this context, the characterization of metabolites produced by species of the genus *Parengyodontium* is of particular interest. Furthermore, a recent study has highlighted the ability of a strain of *P. album* to degrade polyethylene, thus revealing its strong potential in the field of bioremediation [44].

This review provides an updated summary of knowledge on the secondary metabolites of the genus *Parengyodontium*, focusing on highlighting their chemical diversity, biological functions, and potential for biotechnological exploitation, based on data from the chemical, mycological, and genomic literature.

In order to contextualize the chemical repertoire of *Parengyodontium* spp. in relation to its closest relatives, we expand the scope of this review to include the Cordycipitaceae family, contrasting entomopathogenic parasites with saprobic and mycoparasitic lineages. This comparative approach allows us to ask whether lifestyle predicts chemistry at the family level, in particular whether entomopathogens converge on depsipeptides and quinones involved in virulence, immune modulation, and cadaver defense, while saprobes/mycoparasites favor aromatic polyketides and antibiofilm/antimicrobial scaffolds suited to competition for substrates. We then return to *Parengyodontium* as a central genus to assess how its metabolic profile and emerging bioremediation potential fit into the trends at the family level.

## 2. The Chemical Diversity of Cordycipitaceae Family: Comparison Between Entomopathogenic and Saprobic/Mycoparasitic Lineages

The family Cordycipitaceae (Hypocreales) encompasses a broad ecological spectrum dominated by fungi that parasitize insects and spiders, as well as less common saprobic and mycoparasitic lineages. This ecological diversity, clarified by modern phylogenetic revisions and the resolution of several “*Isaria*-type” complexes, provides a solid framework for determining whether lifestyle predicts chemistry at the family level [12,57,58]. In short, entomopathogens tend to converge on depsipeptides and quinones that facilitate infection, immune evasion, and cadaver defense against microbial competitors, while saprobes and mycoparasites often favor aromatic polyketides and antibiofilm/antimicrobial structures suited to substrate competition and niche colonization [22,59,60,61].

Among entomopathogens, *Beauveria bassiana* has become a benchmark for establishing a link between specific metabolites and their function. The cyclooligomer depsipeptide “bassianolide” is a well-characterized virulence factor whose non-ribosomal peptide synthetase (NRPS) biosynthetic pathway has been elucidated; its insecticidal and membrane properties illustrate the direct contribution of depsipeptides to pathogenesis [62]. Similarly, the ionophore beauvericin and the dibenzoquinone oosporein illustrate complementary strategies: oosporein is tightly regulated in a cascade that is activated after the death of the host to curb bacterial growth and secure nutrient resources, and it also participates in immune modulation during infection [63,64,65,66,67,68]. These metabolites underline not only insect pathogenicity but also biocontrol performance, as reflected in studies on nematicidal activity and formulation/production strategies that exploit *Beauveria* toxins and spores [67,69]. Overall, the recurrent appearance of depsipeptides and quinones suggests a conserved “entomopathogenic core in the chemistry of Cordycipitaceae [59,61].

The *Cordyceps* lineage, represented by *Cordyceps militaris* adds a complementary layer focused on nucleoside metabolism. The emblematic cordycepin (3′-deoxyadenosine) lies at the intersection of metabolism and signaling, with a growing understanding of its biosynthesis, transcriptional regulation, and strain engineering for production [70,71,72,73,74,75]. Although cordycepin is not unique to *Cordyceps*, the genus has become an operational model for linking nucleoside biosynthetic gene clusters (BGCs) to metabolite production and bioactivity. Beyond cordycepin, *Cordyceps militaris* produces militarinones, xanthones, and polysaccharides, confirming that even in parasites, the repertoire extends beyond canonical depsipeptides and quinones [70,71,72,74]. Recent genomic and chemical syntheses of *Hypocreales* further emphasize that the diversity and regulation of BGCs, rather than the simple number of genes, best explain the diversity of metabolites at the genus and species levels [60,76].

Other entomopathogenic genera, notably *Akanthomyces* and *Lecanicillium*, illustrate how ecological diversity and cryptic speciation complicate the simple rules of chemistry and lifestyle. Modern systematics has determined species diversity and host associations in *Akanthomyces*, while molecular dating and phylogenetics have refined the classification of *Lecanicillium* within the Cordycipitaceae [77,78]. Although their metabolomes are less extensively mapped than those of *Beauveria* or *Cordyceps*, reports on phenopicolinic acid derivatives, polyketides, and peptide skeletons suggest a common set of entomopathogenic tools, with genus-specific features likely determined by host range and regulatory wiring [60,61,78]. Arachnid-parasitizing lineages such as *Gibellula* add an additional nuance: pigmentosins exhibit antibiofilm properties, thus addressing the need to manage the arthropod-associated microbiome during infection, while *Conoideocrella luteorostrata* produces glycosylated xanthones and cyclodepsipeptides, demonstrating that quinones and xanthones are not limited to saprobes [79,80,81].

In contrast, saprobic and mycoparasitic taxa often emphasize aromatic polyketides and antimicrobial functions compatible with life on heterogeneous substrates or at the expense of other fungi. *Simplicillium*, a mycoparasite of plant pathogens (e.g., powdery mildews), illustrates this phenomenon well. Taxonomic syntheses and species descriptions document a genus adapted to fungal hosts, while genomic analyses of *Simplicillium aogashimaense* reveal an expansion of cell wall-targeting enzymes and an inventory of NRPS/PKS-type BGCs, suggesting antimicrobial and antibiofilm chemistry rather than classical insecticidal virulence factors [31,82,83]. Field applications, such as the antagonism of coffee leaf rust by *Simplicillium lanosoniceum*, correspond to this chemical and ecological profile [84]. Similarly, *Parengyodontium*, which is ecologically nonspecific and often saprobic, has produced anthraquinone-rich polyketide families (engyodontochones A-F) and xanthoquinodine JBIR-99, which has antiproliferative activity, together illustrating a bias towards aromatic polyketides with antimicrobial/antibiofilm and cytotoxic properties [50,55]. It should be noted that *P. album* can oxidatively degrade polyethylene in the marine environment, highlighting a metabolism adapted to recalcitrant substrates and environmental competition rather than host exploitation [44].

Do parasites and saprobes “share the same chemical repertoire”? Current evidence supports a partial overlap framed by lifestyle-specific accents. Entomopathogens repeatedly deploy depsipeptides and quinones for invasion, immune manipulation, and cadaver defense, with nucleoside chemistry (*Cordyceps*) as a distinctive axis; saprobes and mycoparasites are enriched in aromatic polyketides and antibiofilm/antimicrobial functions aligned with substrate competition. Exceptions are instructive rather than contradictory: xanthones and anthraquinones are present in entomopathogens (*Gibellula*, *Blackwellomyces*), and peptide structures appear outside strictly parasitic taxa, indicating modular evolution of the BGC and ecology-dependent regulation rather than rigid partitions [59,60,61,79,80,81] (Table 1). A pragmatic synthesis for the family therefore consists of mapping (i) the dominant compound classes by genus, (ii) the ecological function during the life cycle, and (iii) the context/regulation of the BGC, and then verifying whether changes in host range or substrate are reflected in predictable chemical transitions within the Cordycipitaceae.

Currently, 511 genomes from Cordycipitaceae family have been sequenced and are available in the database but only 6 (*Cordyceps militaris* (ATCC 34164), *Beauveria bassiana* (HN6), *Cordyceps militaris* (CH1), *Cordyceps javanica* (Apopka 97), *Cordyceps gunnii* (Cg-01), *Cordyceps javanica* (Bd01)) have been fully assembled. Building on the genome metrics summarized in Table 2 (assembly size, GC content, number of genes), we align genomic potential with the chemical profiling by analyzing representative genomes using fungiSMASH v8.0.4. We detected 33–51 biosynthetic regions per genome, with NRPS-type clusters predominating across entomopathogenic taxa and PKS and terpene classes represented in all analyzed genomes (Table 3). Hybrid regions involving ≥2 families (NRPS/PKS/terpene) were frequent (6–7 per genome), underscoring combinatorial biosynthetic potential. For *Lecanicillium saksenae*, *Simplicillium aogashimaense*, *Parengyodontium torokii*, *B. bassiana* (HN6), *C. militaris* (CH1), *C. gunnii* (Cg-01) and *C. javanica* (Bd01) annotated assemblies suitable for fungiSMASH were not available; we therefore restrict quantitative comparisons to *Beauveria bassiana* (ARSEF 2860), *Cordyceps militaris* (CM01), *Akanthomyces muscarius* (Ve6), *Cordyceps javanica* (Apopka 97) and *Cordyceps militaris* (ATCC 34164).

## 3. The Chemical Diversity in Parengyodontium Genus (Focus Section)

### 3.1. Identified Secondary Metabolites Classified by Chemical Family

Fungal species of the *Parengyodontium* genus synthesize a variety of secondary metabolites, mainly polyketics, terpenes and alkaloid derivatives, as well as other compounds. The identification of these molecules is carried out by extensive spectroscopic analyses, including mass spectrometry (MS and MS/MS) and nuclear magnetic resonance (NMR) [51,85].

#### 3.1.1. Polyketides

Polyketides constitute a major class of secondary metabolites isolated from bacteria, fungi, plants, insects, mollusks, sponges, algae, lichens, and crinoids [86]. These molecules, characterized by their biosynthesis via polyketide synthase (PKS) pathways, exhibit structural and functional diversity [50,85].

**Figure 4:**
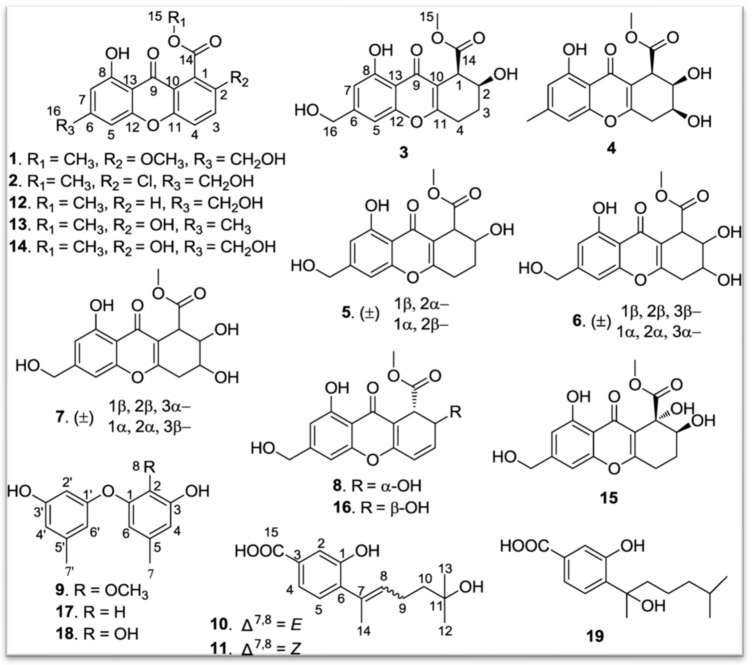
Structures of compounds **1–19** [84]. (Image reproduced/adapted from previously published works as cited.)

- Chromone: *P. album* DFFSCS021, isolated from deep marine sediments, synthesizes eight new chromones, named Engyodontiumones A–H (1–8) [85] (Figure 4).
- Phenolic derivatives and benzoates: Three new phenolic derivatives were isolated from *P. album* DFFSCS021. These include Engyodontiumone I (10) and J (11) as well as 2-methoxyl cordyol C (9) [85] (Figure 4).

**Figure 5:**
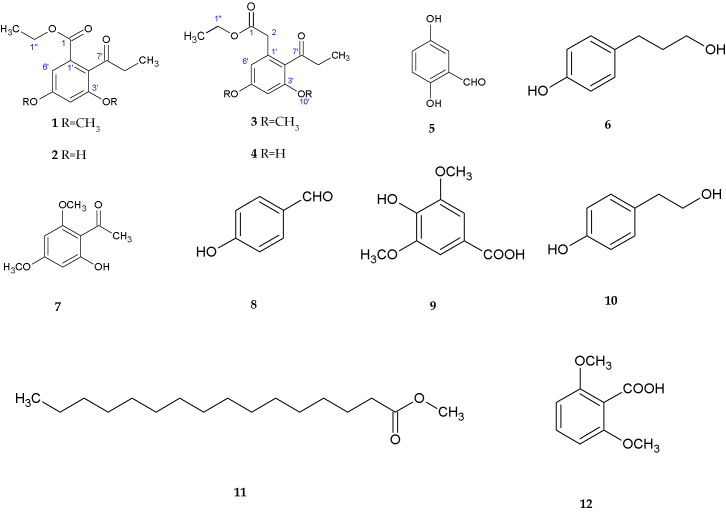
Structure of benzoate derivatives (**1** and **2**) and phenylacetate derivative (**3**) [56]. (Image reproduced/adapted from previously published works as cited.)

In addition, two new benzoate derivatives, ethyl 3,5-dimethoxy-2-propionylbenzoate and ethyl 3,5-dihydroxy-2-propionylbenzoate (1 and 2 in Figure 5, respectively), as well as a phenylacetate derivative, ethyl 3,5-dimethoxy-2-propionylphenylacetate (3 in Figure 5), were discovered in a strain of *Parengyodontium album* from deep marine sediments [56].

- Other polyketides: Molecules such as sydowinine A (12), pinselin (13), sydowinine B (14), aspergillusone B (15), AGI-B4 (16), diorcinol (17), cordyol C (18), and hydroxysydonic acid (19), also classified as polyketides, have been identified in *Engyodontium album* [85] (Figure 4).

**Figure 6:**
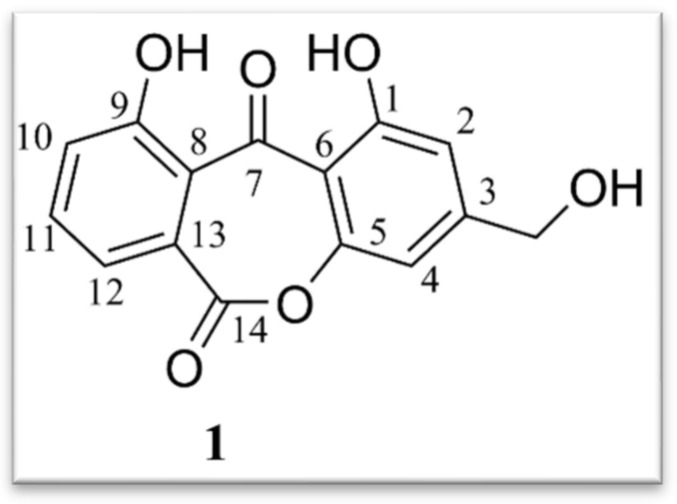
Structure of Alternaphebol B2 [51]. (Image reproduced/adapted from previously published works as cited.)

In *P. album* SCSIO SX7W11, a species isolated from corals, a new aromatic polyketide, alternaphenol B2 [51], was isolated (Figure 6).

#### 3.1.2. Terpene Compounds

Although less prevalent than polyketides, terpenes have been identified in species of the genus *Parengyodontium*. Terpenes are a class of natural organic compounds consisting of isoprene units (C5H8), biosynthesized mainly by plants and many microorganisms.

- Cytochalasin: Genomic analysis of *Parengyodontium torokii* predicted the biosynthesis of cytochalasine K, a terpenoid compound [29]. This metabolite was identified by LC-MS in a fungal extract, confirming the in silico predictions [29]. Cytochalasin compounds are known for their structural diversity and biological activities, particularly anti-cancer activities [87].
- Other terpenes: Gene clusters for the production of other terpenes or related compounds such as squalestatin S1 have been identified in the species *P. torokii* [29].

Steroids, which are tetracyclic compounds derived from the cyclization of triterpenes, including 5,8-epidioxyergosta-6,9(11),22-trien-3-ol and rtgosta-4,6,8(14), 22-tetrean-3-ol, have also been identified in *P. torokii* [29].

#### 3.1.3. Alkaloids and Other Chemical Families

In addition to polyketides and terpenes, fungi of the genus *Parengyodontium* synthesize other classes of secondary metabolites, including alkaloids and various other compounds.

**Figure 7:**
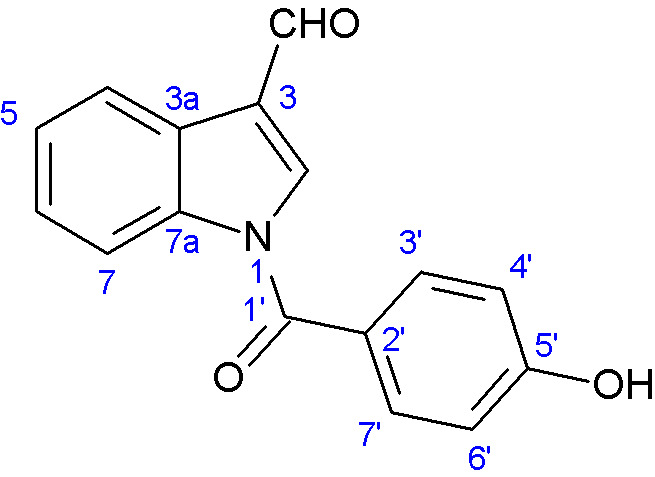
Structure of 1-(4-hydroxybenzoyl)indole-3-carbaldehyde [52]. (Image reproduced/adapted from previously published works as cited.)

**Figure 8:**
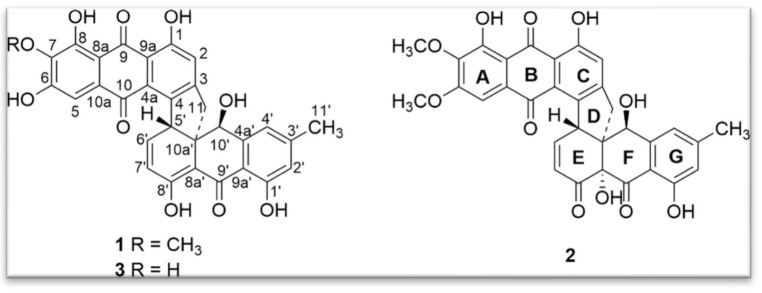
Structure of parengyomarin A (**1**), parengyomarin B (**2**) and torrubiellin B (**3**) [42]. (Image reproduced/adapted from previously published works as cited.)

- Indole alkaloids: A new indole alkaloid, 1-(4-hydroxybenzoyl)indole-3-carbaldehyde (Figure 7), was isolated from a strain of *Engyodontium album* derived from a marine sponge [52]. Alkaloids are a family of nitrogen-containing compounds known for their major pharmacological properties. They include, but are not limited to, morphine (analgesic), quinine (antimalarial), atropine (anticholinergic), etc.
- Torrubiellin derivatives: A strain of *P. album* isolated from the leaves of *Avicennia marina* (in mangroves) produces new torrubiellin derivatives, named parengyomarin A (1) and B (2), in addition to the already known torrubiellin B (3) (Figure 8). Other compounds such as emodin and emodic acid have also been identified in extracts of this fungus [42].
- Other compounds: Metabolomic analysis of *P.torokii* identified several other molecules, including cyclic peptides such as cyclo(L-Leu-L-Pro) and (3β,22E)-cyclo(L-Pro-L-Leu), fatty acids (6,9-octadecadienoic acid), and compounds such as cephalochromin and betulinan [29]. In silico predictions have also suggested the presence of equistetin, cephalosporin C, EQ-4, curcupallide-B, pyranonigrin E and dimethylcoprogen [29].

## 4. Potential Applications of Secondary Metabolites Produced by *Parengyodontium* spp.

Secondary metabolites produced by fungi of the genus *Parengyodontium* have potential applications in several industrial sectors, particularly pharmaceuticals and agri-food, due to their diverse biological activities (Table 4):

- Anticancer activities: Polyketides, such as Engyodontiumones, have shown selective cytotoxicity against the human histiocytic lymphoma cell line U937, with IC50 values of 4.9 and 8.8 µM for compounds 8 and 16, respectively [85]. Cytochalasin K, identified in *P. torokii*, has been shown to influence the final stages of mitosis and have a marked synergistic effect on cancer cells [29]. Cytotoxic polyketides (Xanthoquinodin JBIR-99) have been isolated from *Parengyodontium album* [50]. Alternaphenol B2 from *P. album* showed selective inhibitory activity against mutant isocitrate dehydrogenase R132H (IDH1m), a relevant target for cancer treatment, with an IC50 of 41.9 µM [51].
- Antibacterial activities: Several metabolites exhibited antibacterial properties. Compounds 8, 15, and 16 from *P. album* showed moderate antibacterial activity against *Escherichia coli* and *Bacillus subtilis* [85]. A phenylacetate derivative (compound 3) from *P. album* exhibited inhibitory activity against methicillin-resistant *Staphylococcus aureus* (MRSA) and *Vibrio vulnificus*, with MICs of 7.8 and 15.6 µg/mL, respectively [56]. Torrubielline derivatives have also demonstrated antibacterial activities [42]. Fungal mycelium extracts possess antimicrobial properties, with superior efficacy against Gram-positive bacteria [54].
- Antilaryngeal activities: Compound 15, a polyketide from *P. album* DFFSCS021, showed potent antilaryngeal activity against the establishment of barnacle larvae (*Balanus amphitrite*) [85]. This property suggests potential for the development of biofoulants.
- Enzymes and other applications: Genomic analyses of *P. torokii* have revealed the presence of enzyme families such as GH33 glycosyl hydrolases (sialidases) and GT20 and GT34 glycosyltransferases. These enzymes may have biotechnological applications, particularly for the modification of glycoconjugates or the biosynthesis of disaccharides and oligosaccharides [29]. In addition, the genus *Parengyodontium* is of interest in bioremediation, as evidenced by *P. album*, which is capable of biodegrading certain synthetic plastics such as polyethylene [44]. This result suggests the presence of enzymes such as laccases, oxidases, and peroxidases [44]. Laccases are multi-copper oxidases widely found in fungi, plants and bacteria. Fungal laccases are particularly valued because they oxidize a wide range of phenolic and non-phenolic substrates (often with redox mediators) while reducing O_2_ to H_2_O, enabling applications ranging from lignin modification to green synthesis and pollutant removal [88,89]. Peroxidases, including lignin peroxidase (LiP), manganese peroxidase (MnP), versatile peroxidase (VP), and dye-decolorizing peroxidases (DyPs), are heme enzymes that use H_2_O_2_ to attack lignin and recalcitrant aromatic compounds. Recent work highlights engineered VPs, MnP-mediated oxidation via Mn^3+^ chelates, and DyP diversity across fungi for lignin/dye transformation [90,91,92]. Oxidases generate H_2_O_2_ from O_2_. An example is glucose oxidase, which oxidizes β-D-glucose to D-glucono-δ-lactone and H_2_O_2_ and remains central in biosensors and food applications [93,94,95]. In white-rot systems, aryl-alcohol oxidase supplies H_2_O_2_ to ligninolytic peroxidases and can also act as a quinone reductase, enhancing the degradation of lignin by peroxidases [96,97].

## 5. Discussion: Perspectives, Limitations and Future Directions

At the family level, Cordycipitaceae show partial overlap in their chemical repertoires, with specificities linked to their lifestyle: entomopathogens regularly use depsipeptides and quinones, while saprobes/mycoparasites are rich in aromatic polyketides and antibiofilm functions. In this context, *Parengyodontium* aligns with the saprobic/mycoparasitic pole (anthraquinone-rich polyketides and antibiofilm activity), while its oxidative degradation of polyethylene highlights an adaptation to a niche that goes beyond host exploitation. This framework refines our interpretation of *Parengyodontium* chemistry and identifies testable predictions for gene cluster regulation across the family.

Current data highlight the value of fungal species of the genus *Parengyodontium* as producers of secondary metabolites. The chemical diversity observed, ranging from polyketides to alkaloids and terpenes, is accompanied by a range of relevant biological activities, including antimicrobial and anticancer properties. These observations confirm the potential of these fungi for the discovery of new molecules of pharmaceutical and biotechnological interest. The prediction of biosynthetic gene clusters, such as those associated with cytochalasine K in *P. torokii* [29], highlights the value of genomic approaches in guiding metabolite research. In addition, the recent discovery of the polyethylene degradation potential of *P. album* suggests strong potential for bioremediation [44]. Beyond its widespread presence on heritage substrates, it has recently been demonstrated that *P. album* biodegrades polyethylene (PE) in marine conditions, mineralizing UV-pretreated PE into CO_2_ at approximately 0.044% per day and incorporating traces of PE-derived carbon into biomass, proving that this is true catabolism rather than simple surface erosion [44]. This ability places *P. album* among a small group of marine fungi whose ability to degrade plastic has been empirically validated, although photodegradation (UV) is a prerequisite for effective fungal attack on PE [44]. In addition, *P. album* and its close relatives produce robust extracellular hydrolases, including alkaline serine proteases from marine isolates, which function at high pH and salinity, valuable characteristics for waste and seawater applications [98,99,100,101]. Combined with its success on low-nutrient, salt-affected heritage surfaces, these traits highlight an ecological versatility consistent with bioremediation potential of plastics and protein/organic residues [34,48,49]. Among the main enzymes relevant to remediation, alkaline serine proteases produced by marine isolates remain active at high pH and salinity, making them suitable for treating protein-rich effluents and proteinaceous biofilms in coastal/industrial environments [98,99,100,101]. While such proteases do not depolymerize polyethylene, they can reduce organic load and improve access for complementary oxidative/hydrolytic processes, thereby supporting multi-step remediation workflows.

However, limitations remain. Although in vitro activities are frequently reported, in vivo studies are less numerous, making it difficult to fully assess the pharmacokinetics and potential toxicity of these compounds. Metabolite production can vary considerably depending on culture conditions and fungal strains [54], requiring extensive optimization of fermentation protocols for large-scale production. Secondary metabolism in Cordycipitaceae is highly responsive to environmental cues and depends on cultivation parameters and biotic context. Medium composition (notably C/N) and culture format OSMAC (One strain-Many Compounds) can switch on/off distinct clusters and alter titers; co-culture frequently unlocks otherwise silent pathways [5,6,7,10]. Salinity, pH, trace ions, light and temperature further reshape expression programs. In *Beauveria* for example, oosporein is tightly regulated across infection and post-mortem phases, while depsipeptides such as bassianolide and beauvericin contribute to virulence within defined regulatory cascades [62,63,64,67,68]. In *C. militaris*, nucleoside metabolism (the cordycepin/pentostatin module) is strongly condition- and development-dependent, with medium and light cues affecting pathway activity [70,71,72,73,74,75]. Viewed alongside our BGC inventory (Table 3), the environmental levers offer a practical roadmap to activate silent clusters and better align genomic potential with detectable chemistry in this family.

The complexity of structural identification of new molecules represents a challenge. Distinguishing between cryptic species, particularly within *P. album* [33,34], is essential for accurately assigning metabolic profiles. The resistance of certain strains of *P. album* to traditional antifungal agents [33] suggests a need for new antifungals.

Future directions include targeted exploration of the biodiversity of this genus in unexplored environments for the discovery of novel molecules. The integration of multi-omics approaches (genomics, transcriptomics and metabolomics) will allow a more detailed understanding of biosynthetic pathways and the activation of “silent” gene clusters. In-depth studies of the mechanisms of action of the most promising metabolites and comprehensive toxicological evaluations are necessary for their progression to preclinical development. The development of optimized fermentation methods and efficient purification techniques will contribute to the industrial viability of these compounds.

## 6. Conclusions

Fungi of the genus *Parengyodontium* are sources of secondary metabolites with bioactive properties. Polyketides, terpenes, and alkaloids are the main chemical families identified, with significant activity against cancer cells, bacteria, and barnacle larvae. These discoveries open up prospects for applications in the pharmaceutical, agri-food, and biotechnology fields. Further research, combining advances in genomics and metabolomics, is essential to fully elucidate the biosynthetic potential of these microorganisms. Continued exploration of their diversity and applications could lead to the development of new therapies and industrial solutions.

For a comprehensive understanding, further investigations are required, including in vivo studies to validate the properties of the compounds and research on optimizing their production. The identification and characterization of new molecules derived from these fungi will contribute to efforts to develop bioactive natural products.

## Figures and Tables

**Table 1:** Comparative chemical diversity within Cordycipitaceae.

Genus (Representative Species)	Lifestyle	Dominant Metabolite Classes	Flagship Examples	Ecological/Functional Roles	References
*Beauveria* (e.g., *B. bassiana*)	Entomopathogen (insects)	Depsipeptides (bassianolide, beauvericin); Quinones (oosporein)	Bassianolide; Beauvericin; Oosporein	Virulence (membrane-active), immune modulation, cadaver defense; nematicidal/biocontrol effects	[62,63,64,67,69]
*Cordyceps* (e.g., *C. militaris*)	Entomopathogen (insects)	Nucleosides (cordycepin); Xanthones; Polysaccharides; Peptides	Cordycepin; Militarinones (rep.); Xanthones	Host manipulation, signaling/interference; broader bioactivities; production/engineering model	[70,71,72,73,74]
*Akanthomyces*	Entomopathogen (insects/arachnids)	Polyketides; Peptides (putative); Phenopicolinic-type derivatives (reported historically)	Representative polyketides/peptides (var.)	Pathogenesis and competitive interactions on arthropod hosts; genus-level idiosyncrasies	[77]
*Lecanicillium*	Entomopathogen (insects)	Polyketides; Peptides; (chemistry less mapped than Beauveria/Cordyceps)	Representative polyketides/peptides (var.)	Insect infection; potential overlaps with Akanthomyces toolkits	[78]
*Gibellula*	Entomopathogen (spiders)	Anthraquinones; Antibiofilm compounds	Pigmentosins	Antibiofilm/antimicrobial activity during host colonization and microbiome control	[79]
*Blackwellomyces*	Entomopathogen (insects/arachnids)	Bioxanthracenes; Cyclodepsipeptides	Bioxanthracene derivatives; Cyclodepsipeptides	Antimicrobial/cytotoxic activities likely aiding infection and post-host defense	[80]
*Simplicillium*	Mycoparasite (on fungi)	Polyketides; NRPS/PKS-derived antimicrobials (putative)	Genomic BGC inventory (NRPS/PKS); species-level yet emerging	Antagonism of fungal pathogens (e.g., powdery mildew, coffee rust); niche competition	[31,82,83,84]
*Parengyodontium* (e.g., *P. album*)	Saprobe/Opportunistic	Aromatic polyketides (anthraquinones; xanthoquinodines)	Engyodontochones A–F; JBIR-99	Antibacterial/antibiofilm, cytotoxic activities; oxidative polyethylene degradation	[44,50,55]

**Table 2:** Genome metrics of representative Cordycipitaceae genomes (source: NCBI).

Genus (Representative Species and Strain)	Genome Size/GC%/Number of Genes
*Beauveria bassiana* (ARSEF 2860)	33.7 Mb/51.5/10364
*Cordyceps militaris* (CM01)	32.2 Mb/51.5/9651
*Akanthomyces muscarius* (Ve6)	36.2 Mb/53/12347
*Lecanicillium saksenae* (MUC18310)	33.5 Mb/52.2/nd
*Simplicillium aogashimaense* (ZM-2020)	30.3 Mb/49/nd
*Parengyodontium torokii* (FJII-L10-SW-P1)	30.4 Mb/50.5/nd
*Cordyceps militaris* (ATCC 34164)	33.6 Mb/51/9362
*Beauveria bassiana* (HN6)	37.1 Mb/49/nd
*Cordyceps militaris* (CH1)	32.4 Mb/51.5/nd
*Cordyceps javanica* (Apopka 97)	35.1 Mb/53/10519
*Cordyceps gunnii* (Cg-01)	30.3 Mb/54/nd
*Cordyceps javanica *(Bd01)	34 Mb/53/nd

nd: no data.

**Table 3:** fungiSMASH v8.0.4 summary of predicted BGCs in five Cordycipitaceae genomes.

Species (Strain)	Total Regions	PKS (Any)	NRPS (Any)	Terpenes	Hybrids (≥2)
*Beauveria bassiana* (ARSEF 2860)	51	15	25	15	7
*Cordyceps militaris* (CM01)	33	11	18	8	6
*Akanthomyces muscarius* (Ve6)	50	13	28	14	6
*Cordyceps javanica* (Apopka97)	48	18	25	8	7
*Cordyceps militaris* (ATCC 34164)	37	13	19	8	6

Counting rules. PKS(any) = {T1PKS, T2PKS, T3PKS, transAT-PKS, PKS-like, PKS}. NRPS(any) = {NRPS, NRPS-like, NAPAA, isocyanide-NRP}. Terpenes = {terpene, terpene-precursor}. Hybrids were counted when ≥2 families co-occurred in the same region. Methods note. fungiSMASH v8.0.4; inputs = annotated GenBank; KnownClusterBlast & SubClusterBlast enabled; other options default. Families were tallied from JSON “products” tags.

**Table 4:** Applications of *Parengyodontium* secondary metabolites.

Anticancer	Antibacterial	Antilaryngeal	Enzymes & Bioremediation
Polyketides (Engyodontiumones): selective cytotoxicity (U937) [85] Cytochalasin K in *P. torokii*: impacts late mitosis; synergy on cancer cells [29] Cytotoxic polyketides: Xanthoquinodin JBIR-99[50] Alternaphenol B2 (*P. album*): IDH1 R132H inhibitor [51]	*P. album* compounds 8, 15, 16: moderate activity vs. *E. coli* & *B. subtilis* [85] Phenylacetate derivative: active *versus* MRSA & *V. vulnificus* [56] Torrubielline derivatives [42] Fungal mycelial extracts [54]	Compound 15 (*P. album* DFFSCS021): anti-settlement of *Balanus amphitrite* (Potential for biofouling control) [85]	*P. torokii* genomics: GH33 sialidases; GT20/GT34 glycosyltransferases [29] *P. album* biodegrades UV-pretreated polyethylene [44]

## Data Availability

No new data were created or analyzed in this study. Data sharing is not applicable.
